# Effectiveness, safety, and factors associated with the clinical success of endoscopic biliary drainage for patients with hepatocellular carcinoma: a retrospective multicenter study

**DOI:** 10.1186/s12876-020-01594-4

**Published:** 2021-01-13

**Authors:** Akihiro Matsumi, Hironari Kato, Toru Ueki, Etsuji Ishida, Masahiro Takatani, Masakuni Fujii, Masaki Wato, Tatsuya Toyokawa, Ryo Harada, Hirofumi Tsugeno, Minoru Matsubara, Hiroshi Matsushita, Hiroyuki Okada

**Affiliations:** 1 Department of Gastroenterology and Hepatology, Okayama University Graduate School of Medicine, Dentistry, and Pharmaceutical Sciences, 2-5-1 Shikata-cho, Kita-ku, Okayama, 700-8558 Japan; 2 Department of Internal Medicine, Fukuyama City Hospital, Fukuyama, Japan; 3 Department of Gastroenterology, Kurashiki Central Hospital, Kurashiki, Japan; 4 Department of Internal Medicine, Japanese Red Cross Society Himeji Hospital, Himeji, Japan; 5 Department of Internal Medicine, Okayama Saiseikai General Hospital, Okayama, Japan; 6 Department of Gastroenterology, Kagawa Prefectural Central Hospital, Takamatsu, Japan; 7 Department of Gastroenterology, National Hospital Organization Fukuyama Medical Center, Fukuyama, Japan; 8 Department of Gastroenterology, Japanese Red Cross Okayama Hospital, Okayama, Japan; 9 Department of Internal Medicine, Tsuyama Central Hospital, Tsuyama, Japan; 10 Department of Internal Medicine, Sumitomo Besshi Hospital, Niihama, Japan; 11 Department of Gastroenterology, Okayama City Hospital, Okayama, Japan

**Keywords:** Endoscopic retrograde cholangiopancreatography, Jaundice, Hepatocellular carcinoma, Liver dysfunction

## Abstract

**Background:**

Only a few reports have assessed the effectiveness of endoscopic biliary drainage (EBD) in hepatocellular carcinoma (HCC) patients with obstructive jaundice and liver dysfunction.

**Methods:**

This was a retrospective study based on the clinical databases from the Okayama University Hospital and 10 affiliated hospitals. All patients received EBD for jaundice or liver dysfunction. The indication for EBD was aggravation of jaundice or liver dysfunction with intrahepatic bile duct (IHBD) dilation. The technical and clinical success rate, complications, factors associated with clinical failure, and survival duration were evaluated.

**Results:**

A total of 107 patients were enrolled in this study. Technical success was achieved in 105 of 107 patients (98.1%). Clinical success was achieved in 85 of 105 patients (81%). Complications related to endoscopic retrograde cholangiography (ERC) occurred in 3 (2.8%) patients. Child–Pugh class C (odds ratio 3.90, 95% confidence interval [CI] 1.47–10.4, *p* = 0.0046) was the only factor associated with clinical failure, irrespective of successful drainage. The median survival duration was significantly longer in patients with clinical success than in those without clinical success (5.0 months vs. 0.93 months; hazard ratio [HR] 3.2, 95% CI 1.87–5.37). HCC Stage I/II/III (HR 0.57, CI 0.34–0.95, *p* = 0.032), absence of portal thrombosis (HR 0.52, CI 0.32–0.85, *p* = 0.0099), and clinical success (HR 0.39, CI 0.21–0.70, *p* = 0.0018) were significant factors associated with a long survival.

**Conclusions:**

EBD for obstructive jaundice and liver dysfunction in patients with HCC can be performed safely with a high technical success rate. Clinical success can improve the survival duration, even in patients expected to have a poor prognosis.

**Trial registration:**

Retrospectively registered.

## Background

Hepatocellular carcinoma (HCC) is the most common cancer worldwide and the most frequent cause of death by cancer. With advances made in the HCC diagnosis and treatment techniques in recent years, the rates of an early tumor diagnosis and long-term survival have been increasing [1–8].

Jaundice is encountered in 5%-44% of patients with HCC at the time of the initial diagnosis and frequently occurs during the later stages of disease [2]. Jaundice and liver dysfunction are usually caused by diffuse tumor infiltration into the liver parenchyma, tumor invasion, and/or progressive terminal liver failure resulting from advanced underlying cirrhosis. Obstructive jaundice caused by tumor invasion or hemobilia is rare in HCC, with an incidence of 0.5%-13% [4], and HCC treatments, such as percutaneous tumor ablation (PTA), arterial embolization, and radiation therapy, rarely cause obstructive jaundice [9]. However, it is closely connected with patients’ mortality as well as jaundice caused by liver dysfunction. Although endoscopic biliary drainage (EBD) is considered useful and effective in patients with obstructive jaundice due to other malignant diseases such as bile duct cancer and pancreatic cancer, only a few studies have investigated the effectiveness of EBD in HCC patients with obstructive jaundice and liver dysfunction or obstructive cholangitis, and most of them have only included a small number of patients [3].

We carried out a retrospective analysis of the outcomes of EBD for obstructive jaundice or liver dysfunction with or without cholangitis caused by HCC in a large number of patients and clarified the factors associated with treatment success and prognosis extension.

## Methods

This retrospective study was based on the clinical databases of the Okayama University Hospital and 10 affiliated hospitals. Data were extracted from the patients who underwent endoscopic stent placement for obstructive jaundice caused by HCC from April 2013 to October 2018. Biliary strictures considered to be associated with therapy for HCC, such as PTA, trans-arterial embolization, and radiation therapy, were omitted. Informed consent was obtained via an opt-out option on the website. The Institutional Review Board of Okayama University approved this study.

### Reference for endoscopic treatment

The indication for endoscopic stent placement was aggravation of jaundice or liver dysfunction with intrahepatic bile duct (IHBD) dilation. Some patients had cholangitis in addition to jaundice or liver dysfunction. Jaundice was defined as a serum total bilirubin concentration over 3 mg/dl, and liver dysfunction was defined as hepatobiliary enzyme elevation compared to the baseline of each patient. IHBD dilatation was defined when an axial scan by computed tomography, magnetic resonance imaging, or ultrasound sonography showed the lumen of the intrahepatic bile duct to be more than 2 mm in diameter.

In accordance with Tokyo Guidelines 2018 [10], cholangitis was defined as the presence of leukocytosis, elevation of C-reactive protein (CRP), or a fever (temperature ≥ 38 °C), in addition to the findings indicating cholestasis and/or the IHBD dilatation previously mentioned.

### Endoscopic procedures

Endoscopic retrograde cholangiography (ERC) was performed using a duodenoscope (TJF260V, JF260V; Olympus Optical Co., Tokyo, Japan) in all cases. After confirming the biliary stricture caused by HCC with ERC, a plastic stent (PS) or endoscopic nasobiliary drainage (ENBD) tube was placed. The stent diameter (5–7 Fr) varied depending on the diameter of the bile duct. The number of PS or ENBD tubes was chosen according to the number of branches divided by the stricture. In cases with several divided branches, a maximum of three stents was placed into the major branches: left hepatic duct, anterior branch of the right hepatic duct, and posterior branch of the right hepatic duct. ENBD was sometimes used for initial drainage for patients with hemobilia due to bleeding of HCC. If ENBD was effective and hemostasis was confirmed, the ENBD tube was replaced with a PS to avoid cholestasis with a clot, even in cases without biliary stricture. Metallic stents were not used due to reports indicating that the survival rate was significantly extended in patients with HCC undergoing endoscopic biliary draining with PSs compared to those treated with metallic stents [7]. In cases in which endoscopic procedures were deemed difficult, percutaneous transhepatic biliary drainage (PTBD) was employed. If clinical success could not be achieved despite successful stent placement, then the type of stent, e.g. straight type or Zimmon type, was changed, and/or the branch into which the PS was placed was changed. In most cases, endoscopic sphincterotomy was not performed in order to maintain the function of the sphincter of Oddi. Endoscopic sphincterotomy was performed when multiple stents were required.

If a blood clot was detected, it was extracted by a basket and balloon catheters. After clinical success was achieved, follow-up ERC and stent exchange were scheduled two to three months post-treatment. In the patient had a poor condition, stents were kept in place without scheduled stent exchange until stent obstruction occurred.

### Evaluations

The outcomes were evaluated on technical success, clinical success, and survival duration. Technical success was defined as successful placement of a PS or ENBD tube in the first session. Clinical success was defined as at least a 30% reduction of jaundice based on liver function test results after technical success. In cases with cholangitis, the resolution of the inflammatory symptoms and laboratory data, in addition to the resolution of cholestasis findings, was defined as clinical success. Clinical failure was defined as an unsuccessful placement of a PS or an ENBD tube or no achievement of the clinical success previously mentioned, irrespective of the successful placement of the PS or ENBD tube. The survival duration was defined as the time from the endoscopic procedure to death.

Adverse events were evaluated according to the ASGE classification of Standards of Practice Committee [11]. Pancreatitis was defined as severe abdominal pain requiring an analgesic and a more than three-fold increase in serum amylase levels.

### Statistical analyses

Statistical analyses were performed using the JMP® 14 software program (SAS Institute Inc., Cary, NC, USA). We used the chi-square test or Fisher’s test to compare proportions and the Mann–Whitney U-test to compare the median values. We performed a univariate analysis to assess the risk of treatment failure using the chi-squared test. The survival rate about clinical factors was evaluated with the Kaplan–Meier method and Cox proportional hazard model for the univariate analysis. To identify clinical factors associated with a long survival, factors with p < 0.05 in the univariate analysis were analyzed using a multivariate Cox model. The threshold for significance was a value of p < 0.05.

## Results

### Patients characteristics

A total of 107 patients underwent EBD. Table 1 summarizes the patient characteristics. The most common causes of HCC were hepatitis C (43%). Child–Pugh class B was seen in about half of all patients (47%). In over half of all patients, the clinical stage was stage IV, the Barcelona Clinic Liver Cancer (BCLC) classification was C and D, and no portal vein invasion was identified. Cholangitis was present in 43 of 106 patients (40%). Prior to EBD, the median serum total bilirubin level in all 107 patients was 6.47 (0.5–31.5) mg/dl. Prior to EBD, the maximum IHBD diameter was 8.0 (3.0–23) mm.

**Table 1:** Patient characteristics

Patients	107
Age (mean ± SD)	71.5 ± 10.1
Sex (%)	
Male/female	76 (71)/31 (29)
Etiology (%)	
HCV/HBV/non-BC	46 (43)/24 (22)/17 (33)
Child–Pugh score (%)	
5/6/7/8/9/10/11/12/13/14/15	15 (14)/9 (8)/19 (18)/15 (14)/17 (16)/19 (18)/10 (9)/2 (2)/0 (0)/0 (0)/1 (1)
Child–pugh score (%)	
A/B/C	25 (23)/50 (47)/32 (30)
HCC stage (%)	
I/II/III/IV	6 (6)/6 (6)/38 (35)/57 (53)
BCLC classification (%)	
0/A/B/C/D	2 (2)/11 (10)/16 (15)/46 (43)/32 (30)
Grade of portal vein invasion	
VP0/1/2/3/4	58 (55)/2 (2)/13 (12)/12 (11)/21 (20)
Cholangitis (%)	
Present/absent	43 (40)/64 (60)
T-bill before EBS (median, mg/dl)	6.47 (0.5–31.5)
IHBD diameter (median, mm)	8.0 (3.0–2.3)

HBV: hepatitis B virus, HCV: hepatitis C virus, non-BC: not hepatitis B and C virus, BCLC: Barcelona Clinic Liver Cancer, T-Bil: Total bilirubin,EBS: endoscopic biliary stent, IHBD: intrahepatic bile duct

### Technical and clinical outcomes

Bile duct stricture was identified in 94 of 107 (88%) patients, and hemobilia was identified in 34 patients (32%) by ERC. Causes of biliary obstruction were tumor invasion or tumor compression by HCC or metastatic lymph-node in 94 patients and hemobilia in 13 patients. Multiple PSs were placed in 32 patients (30%), including 2 in 26 patients, and 3 in 6 patients.

**Fig. 1:**
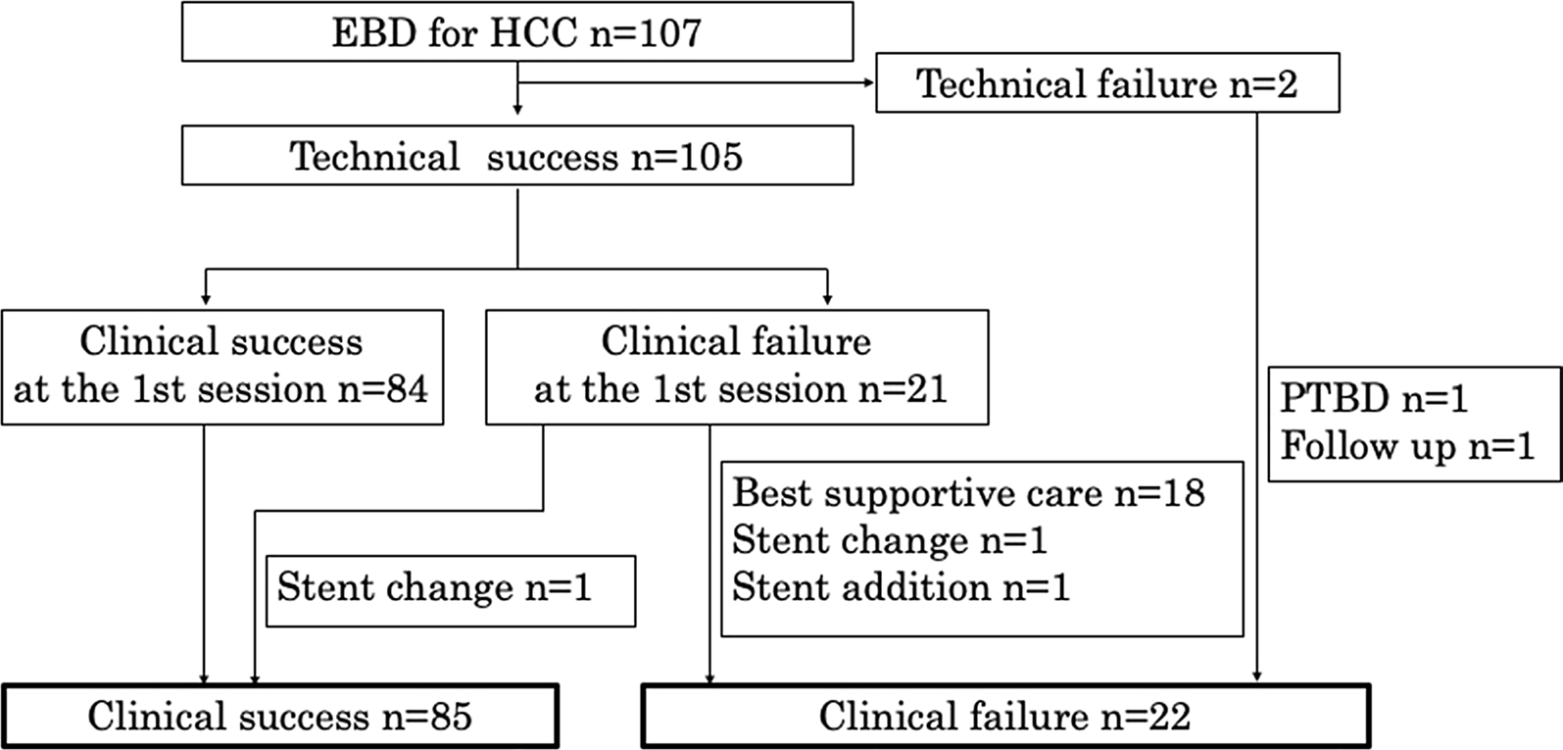
Clinical success was achieved in 85 (81.0%) patients. Most patients who failed to achieve clinical success at the first session were suspected of having liver failure

The patient flow diagram and success rates are shown in Fig. 1. Technical success was achieved in 105 of 107 patients (98.1%) in the first session. The reason for technical failure in the remaining two patients was that the tip of the PS could not pass through the stricture due to the hard nature of the stricture. In these two patients without technical success, no biliary drainage was performed due to liver failure in one patient, whereas PTBD was performed in the other patient, but clinical success still could not be achieved because of liver failure.

In 84 of 105 patients, clinical success was achieved at the 1st session. Of the 21 patients without clinical success, 18 instances were thought to be due to liver failure, so a second session was not performed in these patients. The second session was performed for the remaining 3 patients. The stent was replaced in two patients and added in one, but clinical success was achieved only in one of the patients who underwent stent exchange. The clinical success rate in all patients, including those with and without cholangitis, was 81.0%, 90.2%, and 75%, respectively. An ENBD tube was successfully placed in seven patients. Clinical success was achieved in six of the seven patients, and the ENBD tube was successfully replaced with a PS.

Recurrent biliary obstruction (RBO) occurred in 40 of 85 patients (47%) with clinical success during the observation period (median 96.5 days [IQR 30.5–189.5]). The causes of RBO were debris in 19 (49%), hemobilia in 12 (31%), tumor growth in 5 (13%), and others in 3 (7%).

HCC treatment was performed after endoscopic treatment in 41 of 107 patients. The details of HCC treatment in these patients were trans-arterial therapy in 28 (68%), molecular-targeted drugs in 7 (17%), radiation therapy in 4 (10%) and operation in 2 (5%). Of the 41 patients undergoing HCC treatment, clinical success after endoscopic treatment was achieved in 39.

In 80 of the 107 patients, the survival duration after EBD and causes of death were assessed. The reasons for death were HCC progression in 48 patients (45%), liver failure in 22 (20%), cholangitis in 5 (5%), and other diseases in 5 (5%).

### Adverse events

Complications related to ERC procedures occurred in 3 (2.8%) of the 107 patients. Hyperamylasemia occurred in two patients. Both patients with hyperamylasemia had no abdominal pain and improved with conservative treatment. No post-ERCP pancreatitis occurred. In one patient who underwent ENBD for hemobilia, the total bilirubin level decreased gradually. However, bleeding was out of control, and seven days after ENBD, abdominal pain was reported. The patient was diagnosed with gallbladder hemorrhaging and cholecystitis by computed tomography. Despite receiving intravenous antibiotics, he died eight days after ERCP.

### Clinical success and the survival

Table 2 shows the relationship between patient characteristics and clinical failure after successful EBD. The Child–Pugh class (odds ratio [OR] 4.66, 95% confidence interval [CI] 1.23–30.5, *p* = 0.021) and BCLC classification C/D (OR 3.90, 95% CI 1.47–10.4, *p* = 0.0046) were factors shown to be associated with clinical failure, irrespective of successful drainage in a univariable analysis. In a multivariate analysis, only the Child–Pugh class (OR 2.85, *p* = 0.047) was associated with clinical failure, irrespective of successful drainage.

**Table 2:** Univariate and multivariate analysis of factors associated with clinical success

	Success (n = 85)	Failue (n = 22)	Univariate			Multivariate	
			OR	95% CI	*p* Value	OR	*p* Value
Age (%)				0.48–3.15	0.67		
> 72	43 (81)	10 (19)	1				
≧72	42 (78)	12 (22)	1.23				
Sex (%)				0.15–1.55	0.21		
Male	58 (76)	18 (24)	1				
Female	27 (87)	4 (13)	0.478				
History of radiation therapy (%)				0.21–2.23	0.52		
Present	21 (84)	4 (16)	1				
Absent	64 (78)	18 (22)	0.677				
Child–pugh (%)				0.52–3.48	0.54		0.047
A/B	65 (87)	10 (13)	1			1	
C	20 (63)	12 (37)	3.90			2.85	
Stage (%)				0.16–1.15	0.086		
I/II/III	41 (82)	9 (18)	1				
IV/V	44 (77)	13 (23)	1.35				
BCLC classification (%)							0.175
0/A/B	27 (93)	2 (7)	1	0.73–5.77	0.16	1	
C/D	20 (26)	58 (74)	4.66			2.84	
Portal thrombosis (%)							
Present	36 (75)	12 (25)	1	0.13–1.08			
Absent	49 (83)	10 (17)	0.43				
Biliary structure (%)							
Present	76 (81)	18 (19)	1	0.39–3.00	0.83		
Absent	9 (69)	4 (31)	2.01				
Cholangitis (%)							
Present	37 (75)	6 (14)	1				
Absent	48 (75)	16 (25)	2.06				
Hemobilia (%)							
Present	29 (85)	5 (15)	1				
Absent	56 (77)	17 (23)	1.76				
IHBD diameter (%)							
≦8 mm	44 (73)	16 (27)	1				
> 8 mm	41 (87)	6 (13)	0.40				
Number of stents (%)							
≦1	60 (80)	15 (20)	1				
> 1	25 (78)	7 (22)	1.12				

OR: Odds ratio, CI: Confidential interval, BCLC: Barcelona Clinic Liver Cancer, IHBD: intrahepatic bile duct

**Fig. 2:**
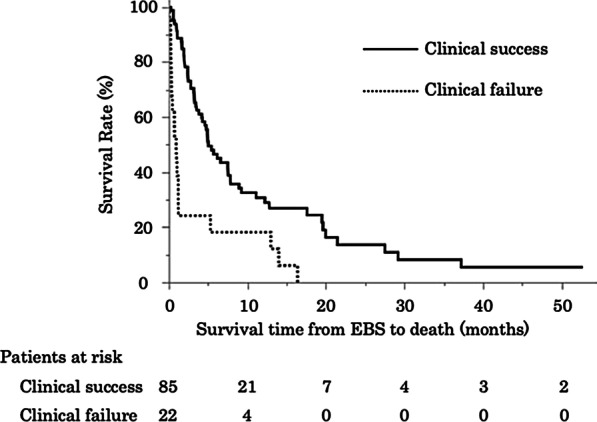
Patients with clinical success had a longer survival than those without clinical success (5.0 months vs. 0.93 months; HR 3.2, 95% CI 1.87–5.37)

The median survival duration was significantly longer in patients with clinical success than in those without success (5.0 months vs. 0.93 months; hazard ratio [HR] 3.2, 95% CI 1.87–5.37) (Fig. 2). In patients with Child–Pugh class C, the survival duration among those with clinical success was longer than among those with clinical failure (2.8 months vs. 0.93 months, *p* = 0.0235).

Table 3 shows the relationship between clinical factors and the survival duration after EBD. The Child–Pugh class (OR 1.70, 95% CI 1.04–2.71, *p* = 0.035), stage (HR 2.35, 95% CI 1.47–3.84, *p* = 0.0005), BCLC classification (HR 2.58, 95% CI 1.51–4.42, *p* = 0.0002), portal thrombosis (HR 0.44, 95% CI 0.27–0.70, *p* = 0.0005), HCC treatment after ERCP (HR 0.32, 95% CI 0.18–0.52, P < 0.0001), and clinical course (HR 3.2, 95% CI 1.87–5.37, P < 0.0001) were associated with the survival duration after EBD in a univariate analysis. The absence of portal thrombosis (HR 0.52, 95% CI 0.28–0.96, *p* = 0.038), HCC treatment after ERCP (HR 0.39, 95% CI 0.21–0.70, *p* = 0.0015), and clinical success (HR 0.39, 95% CI 0.21–0.70, *p* = 0.0018) were significant factors associated with a long survival in a multivariate analysis. In patients with tumor portal thrombosis and without HCC treatment after ERCP, the survival duration among those with clinical success was longer than among those with clinical failure (4.3 months vs. 1.0 months, P < 0.0001; 3.8 months vs. 0.73 months, *p* = 0.0005).

**Table 3:** Univariate and multivariate analysis of clinical factors associated with survival time after endoscopic biliary drainage

		HR	Univariate			Multivariate	
			95% CI	p* value*	HR	95% CI	*p value*
Median age (years)	≦72	1					
	> 72	0.83	0.53–1.30	0.42			
Sex	Male	1					
	Female	0.87	0.52–1.42	0.59			
History of radiation therapy	Present	1					
	Absent	1.02	0.62–1.76	0.95			
Child–Pugh	A/B	1			1		
	C	1.7	1.04–2.71	0.035	1.17	0.64–2.13	0.62
Stage	I/II/III	1			1		
	IV	2.35	1.47–3.84	0.0005	1.53	0.82–2.84	0.18
BCLC classification	0/A/B	1			1		
	C/D	2.58	1.51–4.42	0.0002	1.02	0.40–2.59	0.97
Portal thrombosis	Present	1			1		
	Absent	0.44	0.27–0.70	0.0005	0.52	0.28–0.96	0.038
Biliary stricture	Present	1					
	Absent	0.68	0.30–1.33	0.28			
Cholangitis	Present	1					
	Absent	0.82	0.53–1.30	0.41			
Hemobilia	Present	1					
	Absent	1.48	0.92–2.44	0.11			
Plastic stent	Single	1					
	Multiple	0.71	0.41–1.17	0.18			
HCC treatment after ERCP	Absent	1			1		
	Present	0.32	0.18–0.52	< 0.0001	0.39	0.21–0.70	0.0015
Clinical course	Success	1			1		
	Failure	3.2	1.87–5.37	< 0.0001	2.59	1.42–4.71	0.0018

**Fig. 3:**
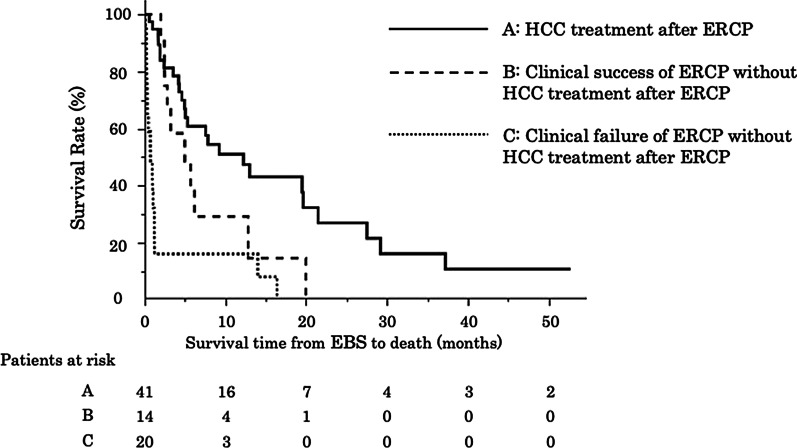
The median survival duration was significantly longer in patients with HCC treatment after ERCP than in those without treatment (12 months vs. 2.8 months; HR 4.3, 95% CI 4.93–21.5). Among patients without HCC treatment after ERCP, the median survival duration was significantly longer in patients with clinical success than in those with clinical failure (3.8 months vs. 0.73 months; HR 2.1, 95% CI 2.8–5.7)

Furthermore, the median survival duration was significantly longer in patients with HCC treatment after ERCP than in those without treatment (12 months vs. 2.8 months; HR 4.3, 95% CI 4.93–21.5) (Fig. 3). Among patients without HCC treatment after ERCP, the median survival duration was significantly longer in patients with clinical success than in those with clinical failure (3.8 months vs. 0.73 months; HR 2.1, 95% CI 2.8–5.7) (Fig. 3).

## Discussion

In this study, we showed that endoscopic biliary drainage for obstructive jaundice in patients with HCC could be performed safely with a high technical success rate. Furthermore, clinical success improves the survival duration, even in patients with poor prognostic factors of HCC, such as Child–Pugh score C, stage IV disease, and tumor portal thrombosis.

The technical success rate of EBD was 98.7% in our study. Reported success rates are 92.1%-100% [2, 3, 7], so our result is not inferior to previously reported findings. In our study, two patients who failed to achieve technical success had hard strictures that a stent could not pass through. Both cases had a history of radiation therapy for HCC. We believe that a hard stricture due to radiation therapy is associated with a risk of treatment failure.

The clinical success rate was 83% in our study. Reported success rates are 35.7%-75.9% [2, 3, 12–15], so our result is superior to that previously reported. The presence of IHBD dilatation is significantly associated with clinical success [3]. However, in our study, an inclusion criterion was the presence of IHBD dilation. Jaundice and liver dysfunction without IHBD dilatation in HCC patients are thought to indicate liver failure, which is difficult to improve via biliary drainage; this may explain why our findings were superior to those of previous studies. However, the clinical success rates of EBD in other malignant strictures, such as hilar cholangiocarcinoma, have been reported to range from 54% to 91.3% [16–19]. Our data are not inferior to those of previous reports on other malignant strictures, either.

Total complication rates reportedly range from 0% to 21.7% [2, 7] in HCC stricture, and 5.3% to 47.8% [19] in other malignant strictures. The complication rate was 2.8% in the present study (hyperamylasemia in 2, gall bladder hemorrhaging in 1), which was similar to the rates in other studies. The rate of post-ERCP pancreatitis (PEP) was reported to be 18.6% [3] for cases of HCC stricture and 0%-38.4% [19, 20] for cases of other malignant stricture. Adler et al. reported that patients with Child–Pugh class C had a higher risk of PEP than others [21]. However, whether or not the risk of PEP in patients with liver cirrhosis is high is controversial [22, 23]. In our study, although there were no cases of post-ERCP pancreatitis, the number of patients was small, so a larger prospective trial is required to resolve this issue.

In our study, Child–Pugh class C was significantly associated with clinical failure. The clinical success rate was 86.7% in cases of class A/B but only 62.5% in class C. In a previous report, the 1-year cumulative survival rates were 45% (Child C), 80% (Child B) and 95% (Child A). Patients with Child–Pugh class C have a poor prognosis because of liver failure [1, 24], so biliary drainage tends to be less effective in these patients than in others. However, on comparing the survival rates between the clinical success and clinical failure cases among patients with Child–Pugh class C, the survival duration in the clinical success cases was longer than that in the clinical failure cases (2.8 months vs. 0.93 months, *p* = 0.0235). In our study, HCC treatment after ERCP was significantly associated with a long survival. In addition, among patients without HCC treatment after ERCP, the median survival duration was significantly longer in patients with clinical success than in those with clinical failure. HCC treatment after ERCP is sometimes difficult in patients who cannot achieve clinical success through endoscopic therapy. Therefore, clinical success with endoscopic therapy is thought to contribute to a long prognosis, both indirectly and directly.

Previous studies have shown that, in addition to successful drainage, subsequent treatment for HCC, the liver function, and HCC tumor stage were significant factors associated with a long survival [2, 3, 8]. Our study found that portal thrombosis and clinical success of ERCP were significant factors influencing the survival according to the multivariate analysis. Indeed, the survival duration in patients with clinical success was significantly longer than in those without clinical success (HR 0.39, 95% CI 0.21–0.70, *p* = 0.0018). Successful drainage may provide HCC patients with jaundice an opportunity for an extended survival, not only through successful drainage itself but also through the opportunity to receive additional HCC treatment. Furthermore, in patients with portal thrombosis who had a poor prognosis, when clinical success was achieved, their survival was extended (HR 0.23, 95% CI 0.11–0.51, *p* = 0.0005).

Several limitations associated with the present study warrant mention. First, all data was reviewed retrospectively. However, this was a multi-center trial, and the number of patients was over 100. To our knowledge, this report included the largest number of patients to date. Second, no data could be analyzed between the survival of the patients with successful biliary drainage in this study and that of the HCC patients without biliary obstruction. Such data would reveal more clearly the effectiveness of biliary drainage in the patients with biliary obstruction with HCC. Third, we used only PSs for biliary drainage. A previous report mentioned that the survival rate was significantly longer in the PS group than in the metallic stent group when patients with HCC underwent endoscopic biliary drainage [7]. Therefore, we used PSs for biliary drainage in this study. However, no study has prospectively compared the effectiveness of biliary drainage using PSs with that using metallic stents in a large number of patients. Randomized controlled trials in large cohorts are required to further investigate this issue. Finally, there may have been some selection bias in our study. When we decided whether or not to perform ERCP for HCC patients, we might have unknowingly excluded patients with a poor condition.

In conclusion, Child–Pugh class C is an important predictor of the effectiveness of endoscopic biliary drainage in HCC patients with liver dysfunction with or without cholangitis. When clinical success is achieved, the prognosis is improved; therefore, we should actively promote biliary drainage for HCC jaundice.

## Conclusions

EBD for obstructive jaundice and liver dysfunction in patients with HCC can be performed safely with a high technical success rate. Clinical success may extend the survival duration, even in patients expected to have a poor prognosis.

## Data Availability

The datasets used and/or analyzed during the current study are available from the corresponding author on reasonable request.

## Abbreviations

HCC: Hepatocellular carcinoma
EBS: Endoscopic biliary stenting
IHBD: Intrahepatic bile duct
CRP: C-reactive protein
ERC: Endoscopic retrograde cholangiography
PS: Plastic stent
ENBD: Endoscopic nasobiliary drainage
PTBD: Percutaneous transhepatic biliary drainage
BCLC: Barcelona clinic liver cancer
PEP: Post-ERCP pancreatitis
